# Cost-Effectiveness of Durvalumab After Chemoradiotherapy in Limited-Stage SCLC

**DOI:** 10.1016/j.jtocrr.2025.100879

**Published:** 2025-07-12

**Authors:** Sheng-Han Tsai, Jui-Hung Tsai, Li-Jun Chen, Szu-Chun Yang

**Affiliations:** a Department of Statistics, College of Management, National Cheng Kung University, Tainan, Taiwan; b Department of Internal Medicine, National Cheng Kung University Hospital, College of Medicine, National Cheng Kung University, Tainan, Taiwan; c Department of Oncology, National Cheng Kung University Hospital, College of Medicine, National Cheng Kung University, Tainan, Taiwan; d National Cheng Kung University Hospital Cancer Center, College of Medicine, National Cheng Kung University, Tainan, Taiwan

**Keywords:** cost-effectiveness, durvalumab, small cell lung cancer, chemoradiotherapy

## Abstract

**Introduction:**

In the ADRIATIC trial, durvalumab consolidation therapy improved the overall survival and progression-free survival of patients with limited-stage SCLC responding to chemoradiotherapy. Based on the data, we aim to assess the cost-effectiveness of the therapy from the perspective of the Taiwanese health care sector.

**Methods:**

Simulated patients with limited-stage SCLC responding to chemoradiotherapy were entered into a partitioned survival model comparing durvalumab consolidation with no consolidation therapy. The model inputs were derived from the ADRIATIC trial (survival outcomes, adverse events, and subsequent therapies), National Health Insurance payments (costs of physician visits, monitoring, drug administration, and end-of-life care), and the hospital cohort (utility values). A lifetime horizon and annual discount rate of 3% were applied. Subgroup, one-way deterministic, and probabilistic analyses were performed.

**Results:**

Durvalumab consolidation therapy incurred an additional $91,734 USD and brought about 0.90 quality-adjusted life years (QALYs) gained, resulting in an incremental cost-effectiveness ratio (ICER) of $101,734 USD per QALY. The ICER remained higher than the willingness-to-pay threshold of $70,000 USD per QALY across most patient subgroups. The most influential factor for the ICER was the cost of durvalumab. If the 4-week drug cost could be reduced to $3893 USD, the ICER would fall below 70,000 USD per QALY. At the willingness-to-pay threshold, durvalumab consolidation therapy had a probability of 0.3% being cost-effective.

**Conclusions:**

Our analysis suggests that durvalumab consolidation therapy is not cost-effective for patients with limited-stage SCLC. Reducing the price of the therapy enhances cost-effectiveness.

## Introduction

Lung cancer is the leading cause of cancer-related deaths worldwide.^1^ SCLC is the most aggressive histologic subtype of lung cancer. Approximately one-third of patients with SCLC are diagnosed at a limited stage.^2^ Definitive chemoradiotherapy with concurrent use of etoposide-platinum and thoracic irradiation has been the standard of care for these patients for the past three decades.^3^ Only recently have there been advances in treatment for limited-stage SCLC. In September 2024, durvalumab, a programmed death-ligand 1 inhibitor, became the first efficacious consolidation immunotherapy for patients with limited-stage SCLC whose disease has not progressed after concurrent chemoradiotherapy.^4^ According to the ADRIATIC trial, durvalumab consolidation therapy, compared with no consolidation therapy, led to longer median overall survival (OS) (55.9 versus 33.4 mo) and progression-free survival (PFS) (16.6 versus 9.2 mo). Following this evidence, durvalumab consolidation therapy was recommended in the National Comprehensive Cancer Network Clinical Practice Guidelines for SCLC.^5^ The U.S. Food and Drug Administration also approved this regimen in December 2024.^6^

Despite the proven clinical benefits of durvalumab consolidation therapy in limited-stage SCLC, maintenance use of this expensive drug imposes profound financial consequences for the health care system. Several previous analyses have evaluated the cost-effectiveness of durvalumab consolidation therapy in patients with stage III NSCLC.^7^, ^8^, ^9^, ^10^, ^11^, ^12^, ^13^, ^14^, ^15^ Evidence suggests that durvalumab consolidation therapy is more cost-effective than no consolidation therapy.^7^, ^8^, ^9^, ^10^, ^11^, ^12^, ^13^^,^^15^ Other researchers have studied the economic outcomes of durvalumab with chemotherapy in the first-line treatment of extensive-stage SCLC and concluded that the immunotherapy and chemotherapy combination is not a cost-effective option.^16^, ^17^, ^18^, ^19^, ^20^ However, no study has explored the cost-effectiveness of durvalumab consolidation therapy in patients with limited-stage SCLC.

On the basis of the published results of the ADRIATIC trial^4^ and considering the costs of drug waste, adverse events (AEs), physician visits, disease monitoring, and end-of-life care, we investigated the cost-effectiveness of durvalumab consolidation therapy versus no consolidation therapy for patients with limited-stage SCLC responding to definitive chemoradiotherapy.

### Model Overview

A partitioned survival model was created to simulate patients with limited-stage SCLC who received durvalumab consolidation therapy or no consolidation therapy after concurrent chemoradiotherapy (Supplementary Fig. 1). Simulated patients with a WHO performance status score of 0 to 1 and without disease progression after concurrent chemoradiotherapy were included in the model. During durvalumab consolidation therapy, patients received 1500 mg of durvalumab every 4 weeks until disease progression or for a maximum of 24 months. Contrastingly, patients in the nonconsolidation therapy group did not receive anticancer therapy until disease progression. After disease progression, patients in both groups received cytotoxic chemotherapy with or without immunotherapy. Etoposide and carboplatin were selected as the subsequent chemotherapy, as most patients in either group had a progression-free interval of more than 6 months,^4^ and retreatment with etoposide-platinum was considered.^5^ The probabilities of subsequent immunotherapy in both strategies were directly derived from the ADRIATIC trial. A model length of 4 weeks was selected for this study because durvalumab was administered every 4 weeks. A lifetime horizon and an annual discount rate of 3% for future costs and life-years were applied. This economic analysis followed the Consolidated Health Economic Evaluation Reporting Standards guideline (Supplementary Table 1).^21^

### Survival Estimates

A web-based software (WebPlotDigitizer; https://automeris.io/WebPlotDigitizer/) was used to extract the PFS and OS curves of durvalumab and placebo groups from the ADRIATIC trial.^4^ The digitized PFS and OS data were translated into imitated patient-level information using the function “digitise ()” in R software.^22^^,^^23^ Parametric models (i.e., loglogistic, Weibull, lognormal, gamma, generalized gamma, exponential) were fit to the data. The model with the most appropriate fit, on the basis of the Akaike Information Criterion or Bayes Information Criterion, was selected. To avoid the logical fallacy of the partitioned survival model, parametric models that produced an extended PFS tail exceeding the OS were excluded. Using the selected parametric models, PFS and OS were extrapolated to the lifetime horizon. Hence, the proportions of patients in the preprogression, postprogression, or death stages at each cycle for both strategies were derived. The modeled PFS and OS curves of the durvalumab consolidation and no consolidation groups were compared with the ADRIATIC trial results.^4^

### Cost and Utility Input

This cost-effectiveness analysis was conducted from the perspective of the Taiwanese health care sector. Direct medical costs, on the basis of payments from the National Health Insurance (NHI), were considered. These included per-cycle costs of physician visits, general monitoring (chest radiography, hemogram, and biochemistry tests), computed tomography, drug administration, and a one-off cost of end-of-life care (Table 1).^24^ A body weight of 70 kg, a body surface area of 1.84 m^2^, and a glomerular filtration rate of 73 mL/min (i.e., a 65-year-old man with a serum creatinine level of 1 mg/mL) were applied to estimate the doses of anticancer drugs. When calculating the costs of intravenous agents, drug waste was considered by rounding up partially used vials (Supplementary Table 2). In addition, the costs attributable to AEs were estimated by multiplying the incidence rate of AEs by the cost of the respective AEs.^25^ We used the 2024 exchange rate for Taiwan dollars to US dollars (USD).^26^

**Table 1:** Model Parameters^a^

Parameter	Value	Range	Distribution	Source
Proportion of patients in the pre/postprogression state, durvalumab consolidation	Time-variant	--	--	ADRIATIC durvalumab’s PFS/OS curves^4^
Proportion of patients in the pre/postprogression state, no consolidation	Time-variant	--	--	ADRIATIC placebo’s PFS/OS curves^4^
Subsequent therapy, durvalumab consolidation				
Cytotoxic chemotherapy	100%	--	--	ADRIATIC trial^4^
Immunotherapy	20.7%	16.6%–24.9%	beta (17, 65)	ADRIATIC trial^4^
Subsequent therapy, no consolidation				
Cytotoxic chemotherapy	100%	--	--	ADRIATIC trial^4^
Immunotherapy	27.2%	21.8%–32.6%	beta (31, 83)	ADRIATIC trial^4^
Cost of physician visit, USD	12	10–14	gamma (100, 0.12)	NHI payment^24^
Monitoring cost per 4 weeks, USD				
General monitoring^b^	28	22–34	gamma (100, 0.28)	NHI payment^24^
CT with contrast	52	42–63	gamma (100, 0.52)	NHI payment^24^
Administration cost, USD	69	56–83	gamma (100, 0.69)	NHI payment^24^
Drug cost per 4 weeks, USD				
Durvalumab	5739	4591–6887	gamma (100, 57.39)	NHI payment
Etoposide	65	52–77	gamma (100, 0.65)	NHI payment
Cisplatin	53	42–63	gamma (100, 0.53)	NHI payment
Carboplatin	266	213–319	gamma (100, 2.66)	NHI payment
Atezolizumab	4163	3330–4995	gamma (100, 41.63)	NHI payment
Cost of end-of-life care,^c^ USD	3027	2421–3632	gamma (100, 30.27)	NHI payment^24^
Health utility				
Preprogression	0.83	0.75–0.92	beta (16.2, 3.3)	EQ-5D^27^
Postprogression	0.72	0.65–0.79	beta (27.3, 10.6)	EQ-5D^27^

CT, computed tomography; EQ-5D, European Quality of Life-Five Dimensions; NHI, National Health Insurance; PFS, progression-free survival; OS, overall survival; USD, US dollars.

a Supplementary Table 3 for parameters of adverse events.

b Chest radiography, hemogram, and biochemistry tests.

c Hospice consultation, home hospice care, and ward hospice care.

On the basis of a hospital cohort’s data using Taiwanese tariffs,^27^ a utility value of 0.83 was applied for patients with limited-stage SCLC receiving immunotherapy in the preprogression state. In the ADRIATIC trial, 86.3% in the durvalumab group and 89.8% in the placebo group used chemotherapy as the subsequent anticancer therapies.^4^ We applied a utility value of 0.72 for patients in the postprogression state receiving cytotoxic chemotherapy.^27^

### Base-Case Analysis

The incremental cost-effectiveness ratio (ICER) was estimated as the incremental cost divided by the quality-adjusted life-year (QALY). The WHO recommends a cost-effectiveness threshold of 1 to 3 times the gross domestic product (GDP) per capita.^28^ Moreover, the willingness of Taiwanese people to pay for a QALY is approximately 1.57 to 2.15 times the per capita GDP.^29^ Thus, the willingness-to-pay (WTP) threshold was set at two times the Taiwanese GDP per capita in 2024, which was 70,000 USD.^30^ The net monetary benefit of each strategy was calculated as the product of QALY and WTP minus the total cost. All analyses were conducted using *R* version 4.4.1 (The R Foundation for Statistical Computing, Vienna, Austria).

### Subgroup Analyses

The PFS and OS curves of patients with or without prophylactic cranial irradiation, undergoing twice-daily or once-daily previous radiotherapy schedules, and receiving carboplatin-etoposide or cisplatin-etoposide as previous chemotherapy differed from those of the overall patient population.^31^ Therefore, PFS and OS data for each subgroup were fitted, and survival estimates and chemotherapy regimens were varied accordingly for the subgroup analyses.

### Deterministic and Probabilistic Analyses

A one-way deterministic analysis was conducted, and a tornado diagram was generated by varying the probabilities of subsequent immunotherapies, costs of physician visits, monitoring, administration, drugs, end-of-life care, health utilities, incidence rates of AEs, and costs for AEs within plausible ranges (Table 1 and Supplementary Table 3). To address the effects of parameter uncertainties, a probabilistic analysis using a Monte Carlo simulation with 1000 iterations was conducted. A cost-effectiveness scatterplot and acceptability curve were also generated.

### Ethics Statement

This model-based cost-effectiveness analysis was approved by the institutional review board of the National Cheng Kung University Hospital (B-ER-114-165). Informed consent was waived as data was obtained from a published literature review and deidentified information.

## Results

### Base-Case Analysis

The modeled PFS and OS curves for patients in the durvalumab consolidation and no consolidation groups are illustrated in Supplementary Figure 2. On the basis of the AIC and BIC of each parametric model (Supplementary Table 4), log-normal distributions were selected. We extrapolated the PFS and OS curves of durvalumab consolidation and no consolidation to a lifetime horizon, as the PFS and OS probabilities of durvalumab did not approach zero in 20 years. The selected PFS and OS curves closely resembled those reported in the ADRIATIC trial, indicating good calibration of the partitioned survival model. The generalized gamma distribution, which produced extended PFS tails exceeding OS tails, was not selected for modeling the PFS curves.

Compared with no consolidation therapy, durvalumab consolidation therapy incurred an additional cost of $91,734 USD and resulted in 0.90 QALY (1.00 life-years) gained (Table 2). Consequently, the ICER was $101,734 USD per QALY (83,194 USD per life-year). The primary driver of the total cost and the cost difference between the two strategies was the drug cost. The costs of physician visits, disease monitoring, AEs, and end-of-life care played only minor roles in the total cost and cost differences. Likewise, preprogression QALY (life-year) constituted the main difference in effectiveness. Postprogression QALYs (life-years) did not differ between the two groups. No consolidation therapy revealed a higher net monetary benefit ($170,729 USD) compared with durvalumab consolidation therapy ($142,114 USD). The incremental net monetary benefit was $28,615 USD.

**Table 2:** Base-Case Results

Strategy	Costs (USD)	Life-Years	QALYs	Cost Per Life-Year (USD)	Cost Per QALY (USD)	Net Monetary Benefit (USD)
No consolidation	Total cost: 40,686 Physician visits: 560 Monitoring: 3731 Drugs: 27,021 Adverse events: 6347 End-of-life care: 3027	Total: 3.83 Preprogression: 2.38 Postprogression: 1.45	Total: 3.02 Preprogression: 1.98 Postprogression: 1.04	--	--	170,729
Durvalumab consolidation	Total cost: 132,420 Physician visits: 721 Monitoring: 4805 Drugs: 115,330 Adverse events: 8537 End-of-life care: 3027	Total: 4.93 Preprogression: 3.36 Postprogression: 1.57	Total: 3.92 Preprogression: 2.79 Postprogression: 1.13	83,194	101,734	142,114

QALY, quality-adjusted life-year; USD, US dollars.

### Subgroup Analyses

Patients receiving prophylactic cranial irradiation, undergoing a twice-daily previous radiotherapy schedule, and using carboplatin-etoposide as previous chemotherapy revealed longer QALYs (life-years) than all other patients (Table 3). Specifically, patients using carboplatin-etoposide as a previous chemotherapy had the lowest ICER of $69,257 USD per QALY ($53,637 USD per life-year), whereas the highest ICER of $156,370 USD per QALY ($141,331 USD per life-year) was noted in patients using cisplatin-etoposide as a previous chemotherapy. Subgroups of patients other than those using carboplatin-etoposide as a previous chemotherapy revealed a lower net monetary benefit of durvalumab consolidation therapy compared with no consolidation therapy.

**Table 3:** Subgroup Analysis

Strategy	Total Costs (USD)	Life-Years	QALYs	Cost Per Life-Year (USD)	Cost Per QALY (USD)	Net Monetary Benefit (USD)
Receipt of prophylactic cranial irradiation						
No consolidation	46,839	4.53	3.57	--	--	203,050
Durvalumab consolidation	139,423	5.63	4.51	83,990	98,817	176,051
No receipt of prophylactic cranial irradiation						
No consolidation	36,201	3.09	2.42	--	--	133,485
Durvalumab consolidation	122,126	4.10	3.23	84,853	106,763	103,897
Twice-daily previous radiotherapy schedule						
No consolidation	57,893	4.70	3.65	--	--	197,385
Durvalumab consolidation	151,175	6.07	4.74	68,060	85,219	180,726
Once-daily previous radiotherapy schedule						
No consolidation	37,275	3.66	2.90	--	--	165,446
Durvalumab consolidation	126,636	4.58	3.67	96,704	115,583	130,204
Previous chemotherapy: carboplatin-etoposide						
No consolidation	40,777	3.84	3.02	--	--	170,870
Durvalumab consolidation	146,941	5.81	4.56	53,637	69,257	172,009
Previous chemotherapy: cisplatin-etoposide						
No consolidation	42,767	3.94	3.10	--	--	173,953
Durvalumab consolidation	125,649	4.52	3.63	141,331	156,370	128,174

QALY, quality-adjusted life-year; USD, US dollars.

### Deterministic and Probabilistic Analyses

**Figure 1:**
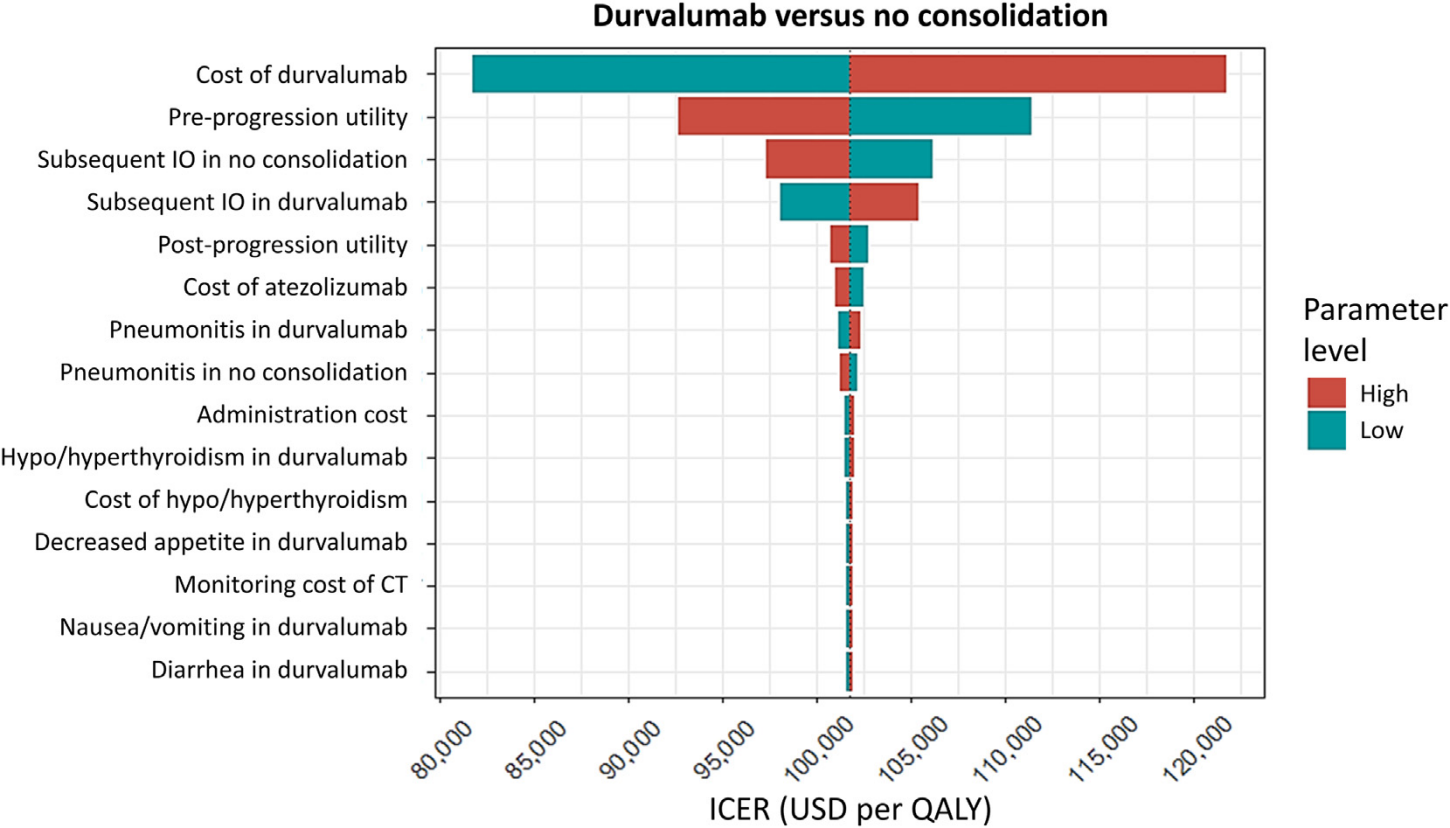
Tornado diagram of durvalumab consolidation therapy versus no consolidation therapy. The dotted line represents the base-case ICER. CT, computed tomography; ICER, incremental cost-effectiveness ratio; IO, immunotherapy; QALY, quality-adjusted life-year; USD, US dollars.

The tornado diagram of durvalumab consolidation therapy versus no consolidation therapy revealed that the cost of durvalumab, the utility value of the preprogression state, and probabilities of subsequent immunotherapy in both strategies were the major determinants of the ICER (Fig. 1). If the 4-week cost of durvalumab could be further reduced to $3893 USD, durvalumab consolidation therapy would become a cost-effective strategy.

**Figure 2:**
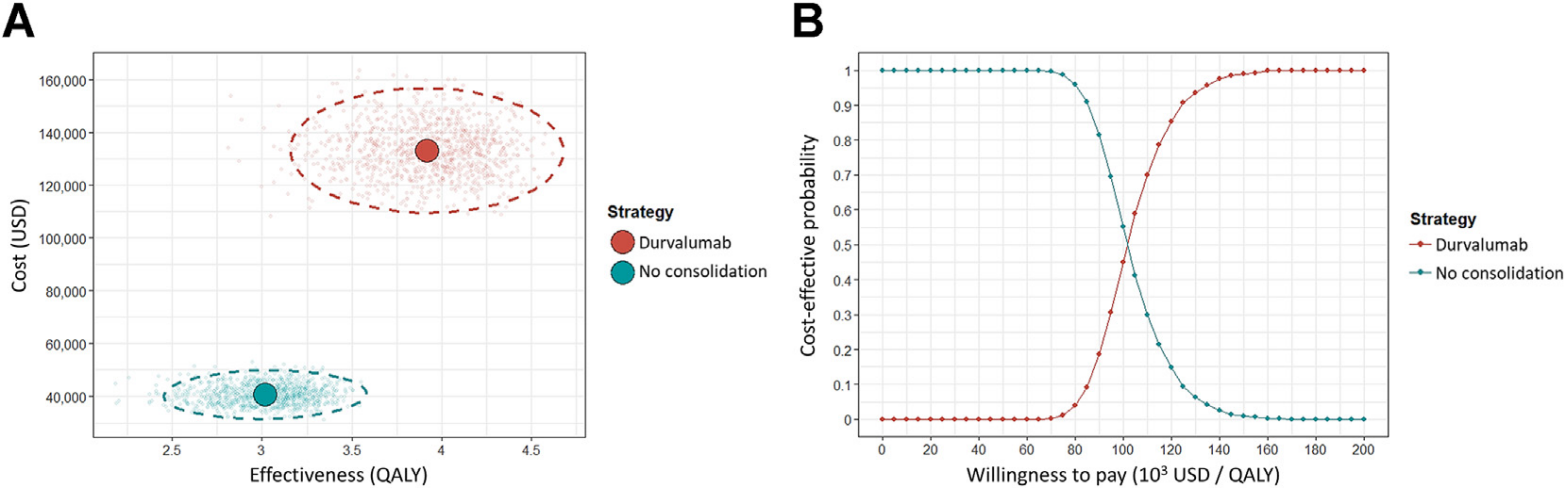
Cost-effectiveness (*A*) scattergram and (*B*) acceptability curves of durvalumab consolidation and no consolidation approaches. The dashed circles in (*A*) indicate the 95% confidence ellipses. QALY, quality-adjusted life-year; USD, US dollars.

The cost-effectiveness scattergram (Fig. 2*A*) illustrates that the 95% confidence ellipse of durvalumab consolidation therapy did not overlap with that of no consolidation therapy. Durvalumab consolidation therapy had probabilities of 0.0%, 0.3%, and 58.8% of being cost-effective at WTP thresholds of $35,000 USD (one per capita GDP), $70,000 USD (two per capita GDP), and $105,000 USD (three per capita GDP) per QALY, respectively (Fig. 2*B*).

## Discussion

This study is the first to evaluate the cost-effectiveness of durvalumab after chemoradiotherapy for patients with limited-stage SCLC. Using a partitioned survival model and directly simulating the PFS, OS, AE, and subsequent therapy data from the ADRIATIC trial (Table 1 and Supplementary Table 3),^4^ we found that durvalumab consolidation therapy was not cost-effective compared with no consolidation therapy from the perspective of the Taiwanese health care sector. Considering the costs of physician visits, disease monitoring, drug waste (Supplementary Table 2), and end-of-life care, the ICER was $101,734 USD per QALY, which exceeds the WTP threshold of $70,000 USD per QALY (Table 2).

In contrast to the cost-effectiveness of durvalumab after chemoradiotherapy in stage III NSCLC,^7^, ^8^, ^9^, ^10^, ^11^, ^12^, ^13^^,^^15^ our study reveals that durvalumab consolidation therapy is not a cost-effective strategy in limited-stage SCLC. Plausible reasons include greater PFS and OS benefits in the PACIFIC study^32^^,^^33^ compared with the ADRIATIC trial.^4^ The hazard ratio for disease progression in the PACIFIC study was 0.52 (95% confidence interval [CI]: 0.42–0.65), whereas that in the ADRIATIC trial was 0.76 (99.816% CI: 0.53–1.08). Similarly, the hazard ratios for death were 0.68 (99.73% CI: 0.47–0.997) in the PACIFIC trial and 0.73 (98.321% CI: 0.54–0.98) in the ADRIATIC trial. Lower PFS and OS benefits in the ADRIATIC trial led to a higher ICER for durvalumab consolidation versus no consolidation in limited-stage SCLC. In addition, the drug cost constituted the main cost difference between the two strategies. In the PACIFIC study, durvalumab consolidation therapy was administered only for a maximum of 12 months, whereas in the ADRIATIC trial, the therapy was offered for a maximum of 24 months. Longer administration of durvalumab in the ADRIATIC trial resulted in considerable drug costs and increased the cost difference between the durvalumab consolidation and no consolidation strategies.

A survival benefit of durvalumab consolidation compared with no consolidation was seen regardless of the receipt of prophylactic cranial irradiation, previous radiation schedule, and previous chemotherapy regimens.^31^ Our subgroup analyses fitting each subgroup’s PFS and OS curves reveal that most of the ICERs exceeded the WTP threshold of $70,000 USD per QALY (Table 3), reinforcing the conclusion that durvalumab consolidation therapy is not cost-effective in limited-stage SCLC. In contrast to real-world clinical practice, patients in the trial using cisplatin-etoposide as a previous chemotherapy experienced the lowest survival benefit of durvalumab consolidation compared with no consolidation.^31^ As a result, durvalumab consolidation therapy is least likely to be cost-effective for these patients.

This study has several policy implications. For example, if the 4-week cost of 1500 mg durvalumab could be further reduced to 3893 USD, reimbursing the drug by Taiwan’s NHI would be feasible (Fig. 1). A trade-off negotiation between drug prices and NHI coverage is indicated. The cost of subsequent immunotherapy in durvalumab consolidation also played a major role in determining cost-effectiveness, emphasizing that readministration of immunotherapy after durvalumab consolidation should be discouraged.

The durvalumab consolidation and no consolidation groups differed significantly in terms of lifetime cost and QALY (Fig. 2*A*). However, the incremental cost still exceeded the monetary benefit of QALY gained. We acknowledge that the WTP thresholds vary across a broad range according to the recommendations of the WHO.^28^ By varying the WTP thresholds over a wide range, durvalumab consolidation therapy would be cost-effective if we were willing to pay a QALY for approximately three times the per capita GDP (Fig. 2*B*).

Although the parametric models with the best fit were selected on the basis of the AIC/BIC, and a generalized gamma distribution was not chosen because it produced extended tails of the PFS curves (Supplementary Fig. 2), our life year estimates were larger than the median survival estimates in the ADRIATIC trial.^4^ The AIC and BIC of Weibull distributions were much higher than those of the selected log-normal distributions (Supplementary Table 4). However, the Weibull distribution is flexible and is typically used to estimate lifelong cancer survival. If the Weibull distributions were adopted to model the PFS and OS curves, the life years of both strategies would decrease and approximate the trial’s estimates of median survival (Supplementary Table 5). Thus, durvalumab consolidation therapy was not cost-effective in terms of cost per QALY gained ($130,264 USD).

Our study has several limitations. First, we applied the same utility value to patients with a preprogression status receiving durvalumab consolidation or no consolidation therapy. Nevertheless, treatment with durvalumab following chemoradiotherapy in limited-stage SCLC did not have a clinically meaningful detrimental effect on patient-reported outcomes.^34^ Thus, the QALY gained from durvalumab consolidation versus no consolidation should not be overestimated. Using the same utility values could even underestimate the ICER. Second, we did not consider postprogression second-line therapy. However, the ADRIATIC trial only reported the probabilities of first-line chemotherapy and immunotherapy after disease progression.^4^ There was also a lack of second PFS information. Synthesizing the second PFS data from another trial (e.g., the IMpower133 study^35^) might introduce bias as it relies on multiple assumptions, such as similar patient characteristics. Finally, PFS and OS curves in the ADRIATIC trial were used to model the lifetime survival of patients. Patients encountered in real-world practice were usually older and had worse Eastern Cooperative Oncology Group performance statuses than those enrolled in the trial. Consequently, the ICER would be underestimated. In addition, we did not consider reduced doses or treatment discontinuation. Effectiveness and cost on the basis of real-world data merit future research.

In conclusion, our analysis suggests that durvalumab after chemoradiotherapy in limited-stage SCLC is not a cost-effective strategy from the perspective of the Taiwanese health care sector. Reducing the price of durvalumab enhances the cost-effectiveness of the therapy. Thoracic oncologists and health policymakers should consider the study’s results when treating patients and reimbursing the drug.

## CRediT authorship contribution statement

**Sheng-Han Tsai:** Data curation, Formal analysis, Investigation, Roles/writing—original draft, Writing—review and editing.

**Jui-Hung Tsai:** Conceptualization, Data curation, Writing—review and editing.

**Li-Jun Chen:** Investigation, Visualization, Writing—review and editing.

**Szu-Chun Yang:** Conceptualization, Data curation, Formal analysis, Investigation, Methodology, Project administration, Resources, Validation, Roles/writing—original draft, Writing—review and editing.

## Disclosure

Dr. Yang reports receiving grants from the 10.13039/100020595National Science and Technology Council and 10.13039/501100004844National Cheng Kung University Hospital during this study. The remaining authors declare no conflict of interest.
