# Supplementary material for: Cost-Effectiveness of Durvalumab After Chemoradiotherapy in Limited-Stage SCLC
Source: JTO Clin Res Rep. 2025 Jul 12;6(11):100879. doi: 10.1016/j.jtocrr.2025.100879 (PMC12605132; doi:10.1016/j.jtocrr.2025.100879)
Supplement: Supplementary Material [file mmc1.pdf]

Supplement

Cost-effectiveness of durvalumab after chemoradiotherapy in limited-stage small-cell lung cancer

Supplementary Figure 1. Model structure. cCRT, concurrent chemoradiotherapy; SCLC, small-cell lung cancer.

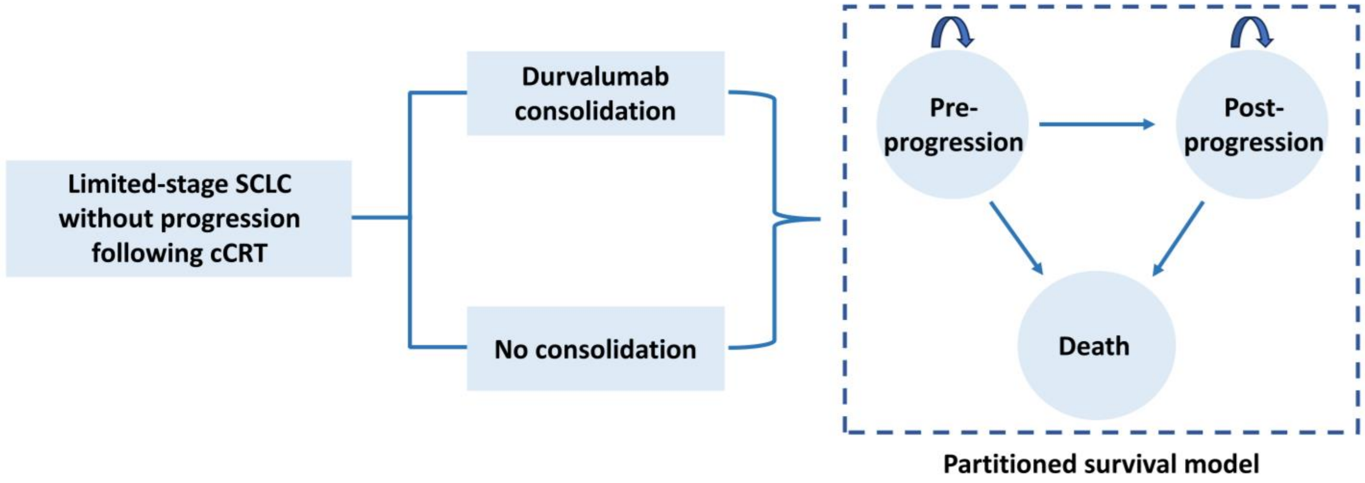

Supplementary Figure 2. Trial (blackened) and modeled (colored) progression-free survival and overall survival curves for patients in the durvalumab consolidation and no consolidation groups. The dashed lines represent the 95% confidence intervals of trial survival curves.

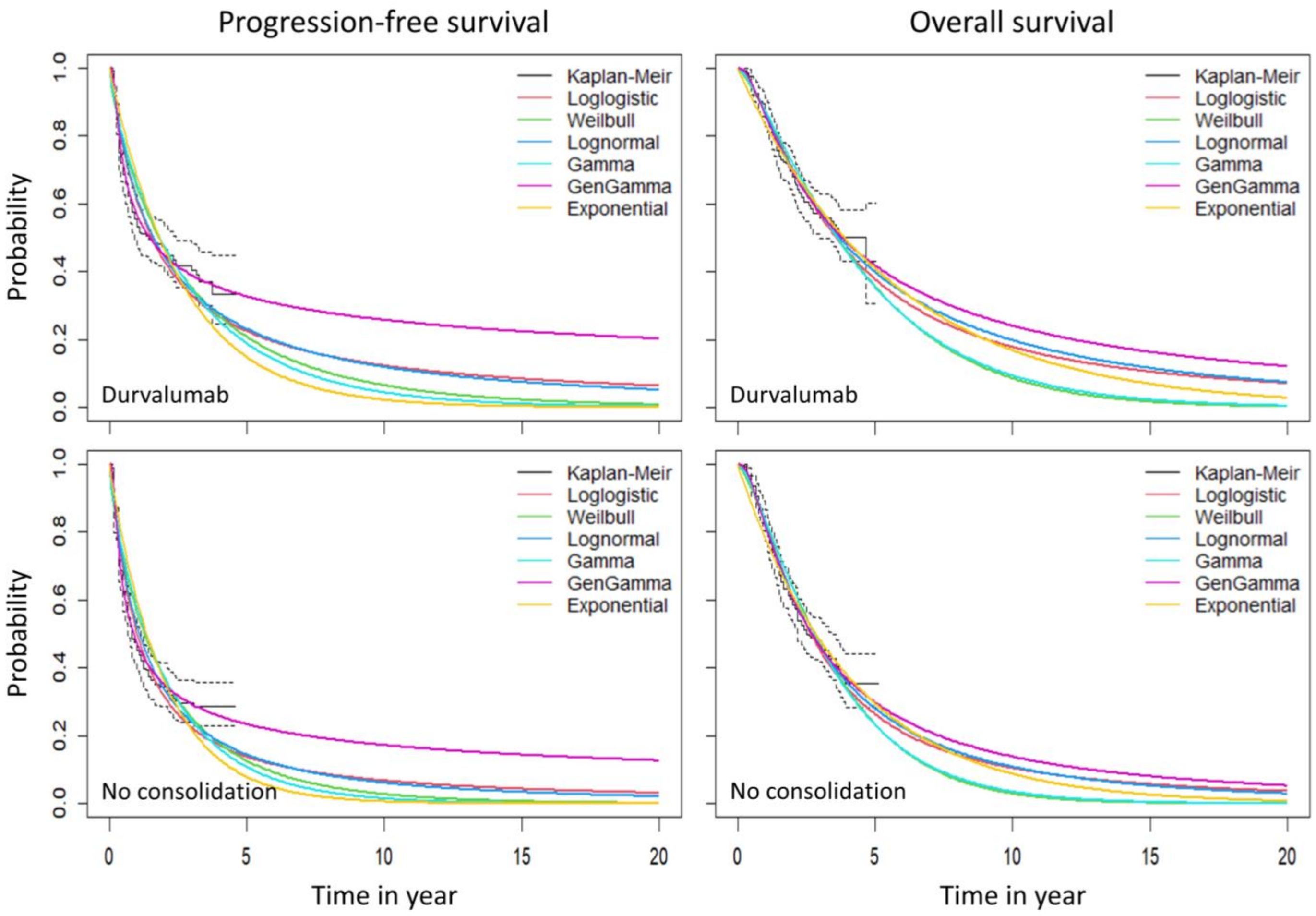

Supplementary Table 1. The CHEERS 2022 checklist

Topic	No.	Item	Location where item is reported
**Title**			
	1	Identify the study as an economic evaluation and specify the interventions being compared.	Title
**Abstract**			
	2	Provide a structured summary that highlights context, key methods, results, and alternative analyses.	Abstract
**Introduction**			
**Background and objectives**	3	Give the context for the study, the study question, and its practical relevance for decision making in policy or practice.	Introduction
**Methods**			
**Health economic analysis plan**	4	Indicate whether a health economic analysis plan was developed and where available.	Materials and Methods, Model overview
**Study population**	5	Describe characteristics of the study population (such as age range, demographics, socioeconomic, or clinical characteristics).	Materials and Methods, Model overview
**Setting and location**	6	Provide relevant contextual information that may influence findings.	Materials and Methods, Model overview
**Comparators**	7	Describe the interventions or strategies being compared and why chosen.	Materials and Methods, Model overview
**Perspective**	8	State the perspective(s) adopted by the study and why chosen.	Materials and Methods, Cost and utility input
**Time horizon**	9	State the time horizon for the study and why appropriate.	Materials and Methods, Model overview
**Discount rate**	10	Report the discount rate(s) and reason chosen.	Materials and Methods, Model overview
**Selection of outcomes**	11	Describe what outcomes were used as the measure(s) of benefit(s) and harm(s).	Materials and Methods, Base-case analysis
**Measurement of outcomes**	12	Describe how outcomes used to capture benefit(s) and harm(s) were measured.	Materials and Methods, Survival estimates & Cost and utility input
**Valuation of outcomes**	13	Describe the population and methods used to measure and value outcomes.	Materials and Methods, Survival estimates & Cost and utility input
**Measurement and valuation of resources and costs**	14	Describe how costs were valued.	Materials and Methods, Cost and utility input, 1st paragraph

Topic	No.	Item	Location where item is reported
**Currency, price date, and conversion**	15	Report the dates of the estimated resource quantities and unit costs, plus the currency and year of conversion.	Materials and Methods, Cost and utility input, 1st paragraph
**Rationale and description of model**	16	If modelling is used, describe in detail and why used. Report if the model is publicly available and where it can be accessed.	Materials and Methods, Model overview
**Analytics and assumptions**	17	Describe any methods for analysing or statistically transforming data, any extrapolation methods, and approaches for validating any model used.	Materials and Methods, Survival estimates
**Characterising heterogeneity**	18	Describe any methods used for estimating how the results of the study vary for subgroups.	Materials and Methods, Subgroup analyses
**Characterising distributional effects**	19	Describe how impacts are distributed across different individuals or adjustments made to reflect priority populations.	Not applicable
**Characterising uncertainty**	20	Describe methods to characterise any sources of uncertainty in the analysis.	Materials and Methods, Deterministic and probabilistic analyses
**Approach to engagement with patients and others affected by the study**	21	Describe any approaches to engage patients or service recipients, the general public, communities, or stakeholders (such as clinicians or payers) in the design of the study.	Not applicable
**Results**			
**Study parameters**	22	Report all analytic inputs (such as values, ranges, references) including uncertainty or distributional assumptions.	Table 1 and Supplementary Table 3
**Summary of main results**	23	Report the mean values for the main categories of costs and outcomes of interest and summarise them in the most appropriate overall measure.	Results, Base-case analysis & Table 2
**Effect of uncertainty**	24	Describe how uncertainty about analytic judgments, inputs, or projections affect findings. Report the effect of choice of discount rate and time horizon, if applicable.	Results, Deterministic and probabilistic analyses & Figures 1 and 2
**Effect of engagement with patients and others affected by the study**	25	Report on any difference patient/service recipient, general public, community, or stakeholder involvement made to the approach or findings of the study	Not applicable
**Discussion**			
**Study findings, limitations, generalisability, and current knowledge**	26	Report key findings, limitations, ethical or equity considerations not captured, and how these could affect patients, policy, or practice.	Discussion
**Other relevant information**			

Topic	No.	Item	Location where item is reported
**Source of funding**	27	Describe how the study was funded and any role of the funder in the identification, design, conduct, and reporting of the analysis	Acknowledgments
**Conflicts of interest**	28	Report authors conflicts of interest according to journal or International Committee of Medical Journal Editors requirements.	Disclosures

Supplementary Table 2. Doses and costs of drugs

Drug	Dose	Unit price[a]	Cost per 4 weeks, USD
Durvalumab	1500 mg every 4 weeks, up to 24 months	1913	5739
Etoposide	100 mg/m^2 * 1.84 m^2 * 3 days every 3 weeks, up to 3 months	8	65
Cisplatin	75 mg/m^2 * 1.84 m^2 every 3 weeks, up to 3 months	13	53
Carboplatin	490 mg (AUC: 5 mg/ml/min) every 3 weeks, up to 3 months	100	266
Atezolizumab	1200 mg every 3 weeks	3122	4163

[a] Durvalumab: 500 mg/vial; etoposide: 100 mg/vial; cisplatin: 50 mg/vial; carboplatin: 450 mg/vial; atezolizumab: 1200 mg/vial.

AUC, area under the concentration-time curve; USD, US dollars.

Supplementary Table 3. Parameter values for AEs of any grade

Parameter	Value	Range	Distribution	Source
Incidence of AE, durvalumab consolidation				
Pneumonitis or radiation pneumonitis	38.2%	30.5-45.8%	beta (100, 162)	ADRIATIC trial[1]
Decreased appetite	16.8%	13.4-20.2%	beta (44, 218)	ADRIATIC trial[1]
Hypothyroidism/hyperthyroidism	16.0%	12.8-19.2%	beta (42, 220)	ADRIATIC trial[1]
Pruritus	13.0%	10.4-15.6%	beta (34, 228)	ADRIATIC trial[1]
Nausea/vomiting	12.6%	10.1-15.1%	beta (33, 229)	ADRIATIC trial[1]
Fatigue	12.2%	9.8-14.7%	beta (32, 230)	ADRIATIC trial[1]
Diarrhea	11.1%	8.9-13.3%	beta (29, 233)	ADRIATIC trial[1]
Rash	10.7%	8.5-12.8%	beta (28, 234)	ADRIATIC trial[1]
Constipation	10.3%	8.2-12.4%	beta (27, 235)	ADRIATIC trial[1]
Anemia	8.8%	7.0-10.5%	beta (23, 239)	ADRIATIC trial[1]
Arthralgia	6.9%	5.5-8.2%	beta (18, 244)	ADRIATIC trial[1]
Hypertension	3.4%	2.7-4.1%	beta (9, 253)	ADRIATIC trial[1]
Incidence of AE, no consolidation				
Pneumonitis or radiation pneumonitis	30.2%	24.2-36.2%	beta (80, 185)	ADRIATIC trial[1]
Decreased appetite	12.8%	10.3-15.4%	beta (34, 231)	ADRIATIC trial[1]
Hypothyroidism/hyperthyroidism	3.8%	3.0-4.5%	beta (10, 255)	ADRIATIC trial[1]
Pruritus	7.2%	5.7-8.6%	beta (19, 246)	ADRIATIC trial[1]
Nausea/vomiting	10.9%	8.8-13.1%	beta (29, 236)	ADRIATIC trial[1]
Fatigue	12.8%	10.3-15.4%	beta (34, 231)	ADRIATIC trial[1]
Diarrhea	8.3%	6.6-10.0%	beta (22, 243)	ADRIATIC trial[1]
Rash	6.0%	4.8-7.2%	beta (16, 249)	ADRIATIC trial[1]
Constipation	9.8%	7.8-11.8%	beta (26, 239)	ADRIATIC trial[1]
Anemia	6.0%	4.8-7.2%	beta (16, 249)	ADRIATIC trial[1]
Arthralgia	10.9%	8.8-13.1%	beta (29, 236)	ADRIATIC trial[1]
Hypertension	1.5%	1.2-1.8%	beta (4, 261)	ADRIATIC trial[1]
Cost for AE (USD)				
Pneumonitis or radiation pneumonitis	6891	5513-8269	gamma (100, 68.91)	NHI claims analysis[2]
Decreased appetite	4223	3378-5068	gamma (100, 42.23)	NHI claims analysis[2]
Hypothyroidism/hyperthyroidism	6217	4974-7460	gamma (100, 62.17)	NHI claims analysis[2]
Pruritus	3642	2914-4370	gamma (100, 36.42)	NHI claims analysis[2]
Nausea/vomiting	5282	4226-6338	gamma (100, 52.82)	NHI claims analysis[2]
Fatigue	4113	3290-4936	gamma (100, 41.13)	NHI claims analysis[2]
Diarrhea	5794	4635-6953	gamma (100, 57.94)	NHI claims analysis[2]
Rash	4233	3386-5080	gamma (100, 42.33)	NHI claims analysis[2]
Constipation	4060	3248-4872	gamma (100, 40.60)	NHI claims analysis[2]
Anemia	5572	4458-6686	gamma (100, 55.72)	NHI claims analysis[2]
Arthralgia	5248	4198-6298	gamma (100, 52.48)	NHI claims analysis[2]
Hypertension	5673	4538-6808	gamma (100, 56.73)	NHI claims analysis[2]

AE, adverse event; NHI, National Health Insurance; USD, US dollars.

Supplementary Table 4. AIC and BIC for each parametric model of progression-free survival and overall survival[a]

Parametric model	Progression-free survival		Overall survival	
	AIC	BIC	AIC	BIC
Durvalumab consolidation				
Loglogistic	484.7	491.9	601.2	608.3
Weibull	504.8	512.0	608.5	615.6
Lognormal	**471.8**	**478.9**	**596.5**	**603.6**
Gamma	509.5	516.6	606.0	613.2
Generalized gamma	432.0[b]	442.7[b]	597.4	608.2
Exponential	513.1	516.7	613.1	616.7
No consolidation				
Loglogistic	488.9	496.1	671.2	678.4
Weibull	524.4	531.6	683.1	690.2
Lognormal	**478.5**	**485.6**	**666.7**	**673.8**
Gamma	531.7	538.8	679.0	686.2
Generalized gamma	434.5[b]	445.3[b]	667.6	678.4
Exponential	536.1	539.7	691.9	695.5

[a] Bold letters denote the AIC and BIC for selected parametric models.
[b] The generalized gamma distributions produced extended tails (Supplementary Fig. 2), indicating logical fallacies, with progression-free survival exceeding overall survival.
AIC, Akaike Information Criterion; BIC, Bayes Information Criterion.

Supplementary Table 5. Sensitivity analysis using Weibull distributions

Strategy	Costs (USD)	Life years	QALYs	Cost per life year (USD)	Cost per QALY (USD)	Net monetary benefit (USD)
No consolidation	Total cost: 33,844<br>Physician visits: 477<br>Monitoring: 3177<br>Drugs: 20,817<br>Adverse events: 6347<br>End-of-life care: 3027	Total: 3.26<br>Pre-progression: 2.15<br>Post-progression: 1.11	Total: 2.58<br>Pre-progression: 1.78<br>Post-progression: 0.80	--	--	147,101
Durvalumab consolidation	Total cost: 129,193<br>Physician visits: 608<br>Monitoring: 4056<br>Drugs: 112,965<br>Adverse events: 8537<br>End-of-life care: 3027	Total: 4.16<br>Pre-progression: 2.90<br>Post-progression: 1.26	Total: 3.32<br>Pre-progression: 2.41<br>Post-progression: 0.91	105,736	130,264	102,990

QALY, quality-adjusted life year; USD, US dollars.

References

1. Cheng Y, Spigel DR, Cho BC, et al. Durvalumab after chemoradiotherapy in limited-stage small-cell lung cancer. *N Engl J Med* 2024;391:1313-1327.

2. Lin CY, Wu TI, Yang SC. Estimating costs associated with adverse events in patients with advanced lung cancer. *Clinicoecon Outcomes Res* 2024;16:761-769.
